# Teachers’ involvement and students’ self-efficacy: Keys to achievement in higher education

**DOI:** 10.1371/journal.pone.0216865

**Published:** 2019-05-24

**Authors:** Sara Ayllón, Ángel Alsina, Jordi Colomer

**Affiliations:** 1 Department of Economics, University of Girona, Girona, Spain; 2 Teaching Innovation Network on Reflective Learning, Institute of Educational Sciences Josep Pallach, University of Girona, Girona, Spain; 3 Department of Specific Didactics, University of Girona, Girona, Spain; 4 Department of Physics, University of Girona, Girona, Spain; University of Westminster, UNITED KINGDOM

## Abstract

We study the relative importance of the three dimensions of need-supportive teaching (NST) and students’ self-efficacy to gain new knowledge about students’ achievement in higher education. NST assumes that teachers are key to the motivation of students, providing autonomy support, structure (support of competence), and involvement (support of relatedness). In turn, self-efficacy raises students’ confidence in their ability to succeed in academic tasks. Drawing on 86,000 records of teaching evaluations by students at the University of Girona (Spain), we present evidence that teachers’ involvement and students’ self-efficacy are the two elements most strongly and positively related to achievement. Students obtain higher marks when they believe that their teachers are dependable and available to offer resources, and when they feel capable of organizing and implementing the courses of action necessary to acquire knowledge. We also find that students’ experience of autonomy support and structure are negatively (or not) correlated with achievement. Subgroup analyses also indicate that students have different needs in different knowledge areas.

## Introduction

Self-Determination Theory (STD) establishes that a learning environment which seeks to improve motivation and achievement should support students’ basic psychological needs for autonomy, competence, and relatedness [1–3]. The need for autonomy stems from the inherent desire that individuals have to be causal agents and to experience volition [3, 4]; the need for competence is associated with individuals’ active tendency toward psychological growth [1, 5]; and the need for relatedness concerns the desire to form and maintain strong and stable interpersonal relationships, to connect with and be accepted by others, and to belong [1, 3, 6]. Moreover, it is impossible to understand aspects of human functioning, such as motivation, learning, or achievement, without considering the role played by self-efficacy beliefs [7]–in our case, the extent to which students feel that they are acquiring the relevant knowledge, skills, and competencies. This paper seeks to understand students’ achievement in higher education, while dealing with both the role played by lecturers’ actions and strategies to nurture students’ basic psychological needs, and the role of students’ efficacy thoughts and beliefs.

In the context of higher education, and within the SDT framework, need-supportive teaching (NST) is a powerful instrument to motivate students and help them achieve better results. Teachers can adopt different motivating styles to respond to students’ psychological needs: *autonomy support*, *competence support* (structure) and *relational support* (interpersonal involvement) [8–10]. First, *autonomy support* gives students the opportunity to reflect on their own needs, resources, and values [3, 11], as well as to express their thoughts, feelings, and perspectives [2, 12]. It implies that teachers provide learning activities in ways that vitalize (rather than neglect or frustrate) students’ inner motivational resources. It can be split into three components [13–15]: teaching is supportive of autonomy, first, when it provides students with choice; second, when it fosters relevance; and third, when teachers show respect, allowing criticism and the use of language that is informational, rather than controlling (which pressures students).

Second, *structure* finds its origin in the students’ experience of effectiveness, and implies that students exercise and extend their capabilities [1, 16]. Teachers can provide structure, for example, by communicating consistent guidelines in a clear fashion. According to [3], structure contains four main components: first, teachers can provide structure by means of clarity, defined in terms of giving clear, understandable, explicit, and detailed instructions, and framing lessons well; second, teachers can offer students guidance in their on-going activities–for example, monitoring their work, or offering help or support when needed; third, teachers can provide students with structure by means of support and encouragement, giving constructive, informational feedback, and thereby making students feel that they have more control over the outcome of their studies; and fourth, teachers can provide students with constructive, informational feedback, thereby helping them to gain control over valued outcomes.

And, finally, *involvement* concerns the desire to form and maintain strong and stable interpersonal relationships [3]. Teachers can be involved by showing affection and interest, by being empathetic, by promoting pro-social behavior in class, by being available to all students, and by showing commitment to students’ learning [12, 17]. Four components of teacher involvement are distinguished [3]: first, teachers can express their involvement by showing affection; second, teachers can express their attunement to the student by showing that they understand him/her; third, teachers can provide resources (e.g. time) to the student; and fourth, teachers can make sure that they are dependable and available to offer support [3].

In addition, the construct *self-efficacy*, introduced by Bandura (1977), is understood as the self-belief a person holds or the personal judgment about his/her competencies [18]. Self-efficacy is primarily a cognitive appraisal of one’s capabilities to fulfill a prospective performance, based on past performances [19]. According to that, self-efficacy focuses heavily on a cognitive component, but is more of a criterion-referenced evaluation of self. In the educational context, students’ perceived self-efficacy is believed to influence the choice of tasks, the level of task performance, the amount of effort put into performing chosen tasks, and the degree of perseverance in task performance [20–22]. According to [23], academic self-efficacy is organized hierarchically, so that students progressively develop differentiated perceptions regarding their capabilities in both large and specific academic domains, as well as skills within these broad domains.

Several meta-analysis studies have shown that autonomy support, structure, and involvement are all positively associated with students’ motivation and engagement [3]. Similarly, student’s self-efficacy has been shown to be a key predictor of academic performance across time, a variety of environments, and different groups [24–26]. However, it is rarely recognized that more studies should focus on the relative importance of each NST dimension (and the cohesion among them), on the one hand, and on self-efficacy, on the other hand, if we hope to get a more nuanced understanding of students’ achievement in higher education. This is something that we undertake in this study. Thus, our objective is to understand the relative importance of the three dimensions of NST and self-efficacy for students’ achievement in higher education, while considering them simultaneously.

## Method

The data used for our empirical analysis consist of the whole universe of teaching evaluations by students at the University of Girona over three consecutive years. Three weeks before their final exams, students answer a brief questionnaire on-line, via the Moodle platform: so students do not know their final mark on the course when they complete the questionnaire. The questionnaire is not compulsory, but students receive messages encouraging them to answer. They can do so at any time of day during the seven days that the questionnaire is on-line, and it is completely anonymous. All lecturers teaching at least 1.5 European Credit Transfer and Accumulation System (ECTS) credits in a subject are evaluated by their students, regardless of the size of the class, and regardless of whether it is a theoretical or a practical course.

In total, we were provided with 86,038 complete students’ records: 27,216 for 2014; 29,946 for 2015; and 28,876 for 2016. We were actually supplied with a larger number of records, but not all students answered all the questions in the questionnaire, and in some instances we were not given the final mark obtained by the student. The available data provide information on 2,204 different course subjects over the period of analysis. In all, 1,832 teachers were evaluated. On average, each teacher is evaluated by nine students per course taught per year.

The teacher-evaluation questionnaire consists of two main parts. In Part A, students are asked (in this order) the following six questions:

1. This teacher has introduced the course syllabus and the evaluation criteria clearly (*STRUC_1*).
2. With this teacher, I learn (*EFFICACY_1*).
3. This teacher motivates me to make an effort and to learn by myself (*AUTON_1*).
4. The course support material that the teacher provides me with helps (*STRUC_2*).
5. The evaluation procedure allows me to show my knowledge (*EFFICACY_2*).
6. This teacher has helped me with my doubts when I consulted him/her (*INVOL_1*).

Finally, Part B simply asks,

7 I evaluate this teacher’s performance as positive.

Questions 1 (*STRUC_1*) and 4 (*STRUC_2*) focus on *structure*. Specifically, these questions refer to the amount and the clarity of the information that teachers provide to students about what is expected and how they can realize those expectations (as, for example, setting rules and providing feedback) [2]. Question 3 (*AUTON_1*) focuses on *autonomy support* as students find themselves more engaged in the process of learning when teachers foster relevance by identifying the value of tasks, lessons, materials, and activities [2, 27]. Question 6 (*INVOL_1*) focuses on teachers’ *involvement* and on the extent to which teachers are available to all students and committed to their learning [28, 29]. Finally, questions 2 (*EFFICACY_1*) and 5 (*EFFICACY_2*) refer to students’ *self-efficacy*, or students’ feelings of competence in relation to their cognitive judgment of their personal capacity to learn [7].

Answers to all questions are on a scale from 1 to 5, where 1 indicates “strong disagreement” and 5 “strong agreement.” Table 1 details the mean and the standard deviation for each question: as shown, the mean hovers around the value of 4, with *STRUC_1* and *INVOL_1* gaining higher values and *AUTON_1* and *EFFICACY_2* receiving the lowest. Over the course of the three years analyzed, we observe no large differences, and the values typically move around the period average. Finally, achievement–which we obtain from the student records–can take any value from 0 to 10, to one decimal point, with a mean of 6.98 and a standard deviation of 1.52. Again, over time these averages remain rather constant. See the supplementary material for further details on the data set used (also Figure in S1 File and Tables A to C in S1 File).

**Table 1:** Mean and standard deviation of the answers to the teaching evaluations.

Question (labelled)	Mean	Std. deviation
STRUC_1	4.31	1.05
STRUC_2	4.06	1.15
AUTON_1	3.98	1.21
INVOL_1	4.32	1.07
EFFICACY_1	4.17	1.14
EFFICACY_2	3.96	1.20

Note. Answers to the questions are on a scale from 1 to 5, whereby 1 refers to “strong disagreement” and 5 to “strong agreement”. *STRUC_1*: This teacher has introduced the course syllabus and the evaluation criteria clearly. *STRUC_2*: The course support material that the teacher provides me with helps. *AUTON_1*: This teacher motivates me to make an effort and to learn by myself. *INVOL_1*: This teacher has helped me with my doubts when I consulted him/her. *EFFICACY_1*: With this teacher, I learn. *EFFICACY_2*: The evaluation procedure allows me to show my knowledge. Data are from teaching evaluations by students at the University of Girona (Spain) from 2014 to 2016. *N* = 86,038 observations.

We base our analysis on linear regression models, where our dependent variable is student achievement (used as a continuous variable from 0 to 10), and our explanatory variables are the scores provided by students in the teaching evaluation questionnaire (also as continuous variables). This allows us to establish whether there is a positive, negative, or non-existent correlation between a student’s perception of how a teacher supports the student’s learning process (in the three dimensions of the NST), self-efficacy and the final mark obtained on the course.

Importantly, all the regressions need to include a series of fixed effects (FE)–that is, dummy variables that control, for example, for course subjects. It is plausible to think that student scores are systematically lower than average in subjects that are (or that are perceived by students to be) more difficult. In that case, it is important to include in the regressions a dummy for each different subject in the data, because that will cancel out the differences in the degree of difficulty *between* subjects, and the results will only exploit variability in students’ answers *within* the same subject. The same is true of course subjects that use different evaluation methods. With the inclusion of fixed effects, we avoid the possibility that our results could be biased because a certain method of evaluation (e.g. essay writing) gives students systematically higher marks than other methods (e.g. final exam). On the other hand, it is also possible that subjects change content or teacher between academic years. Again, in that case, the inclusion of fixed effects by academic year, together with fixed effects by subject, will allow us to explore variability *within* a subject during the same academic year, thus avoiding bias in our results that could come from causes external to our analysis. Finally, a certain number of subjects, particularly in the first year of university, have more than one group class because of the large number of students taking that subject. In those cases, it is important also to include fixed effects by lecturer. That way, our results are drawn from *within* a subject taught by the same teacher during the same academic year. The formal specification of our regression can be found in the supplementary material.

## Results

Table 2 shows the results of the linear regression models on achievement, while considering simultaneously all the answers in Part A of the teacher evaluation questionnaire. Note that we are not interested in the influence of each separate element of the questionnaire on students’ achievement, but rather we want to consider all the elements at the same time. That way, we learn about the relative importance of each dimension within the NST framework and of self-efficacy. Separate regressions by element would always indicate a positive relationship between achievement and higher values of students’ answers. Columns (A) through (D) present the results in a parsimonious manner: without fixed effects at first, and progressively including fixed effects in the rest of the columns. Column (B) considers fixed effects by subject; column (C) by subject and academic year; and column (D) by subject, academic year, and lecturer.

**Table 2:** Results (coefficients) from linear regression models with fixed effects on achievement.

	(A)	(B)	(C)	(D)
STRUC_1	-0.074*** (0.007)	-0.018*** (0.007)	-0.019*** (0.007)	-0.015** (0.007)
STRUC_2	0.029*** (0.007)	0.004 (0.006)	0.004 (0.006)	0.003 (0.007)
AUTON_1	0.021*** (0.008)	-0.007 (0.007)	-0.007 (0.007)	-0.002 (0.007)
INVOL_1	0.064*** (0.008)	0.044*** (0.007)	0.043*** (0.007)	0.043*** (0.007)
EFFICACY_1	0.050*** (0.009)	0.072*** (0.008)	0.072*** (0.008)	0.084*** (0.008)
EFFICACY_2	0.197*** (0.007)	0.124*** (0.006)	0.124*** (0.006)	0.119*** (0.006)
FE subject	No	Yes	Yes	Yes
FE year	No	No	Yes	Yes
FE lecturer	No	No	No	Yes
R-squared	0.046	0.322	0.322	0.343

Note: Standard errors in parentheses. Authors’ elaboration using teacher-evaluation questionnaires and student records at the University of Girona, 2014–2016. *N* = 86,038 observations. Level of significance

*** p<0.01

** p<0.05

* p<0.1.

Column (A) indicates that the more that students are provided with good materials (*STRUC_2*), are motivated by the lecturer (*AUTON_1*), feel that they are learning (*EFFICACY_1*), feel that they are properly evaluated (*EFFICACY_2*), and feel that they are supported when they have doubts (*INVOL_1*), the higher their achievement. As is shown, all the coefficients from *STRUC_2* to *EFFICACY_2* are positive and statistically significant at the 99% confidence level. Only *STRUC_1*, which relates to how clearly the lecturer presented the syllabus, is negatively related to achievement. Importantly, though (and as explained above), these results are likely to be biased, because they do not consider the fact that different subjects, in different academic years, taught by different lecturers, may yield systematically different levels of achievement. Thus, our preferred specifications are presented in columns (B) to (D), which control sequentially for such possible biases.

Only *INVOL_1*, *EFFICACY_1*, and *EFFICACY_2*, in columns (B) to (D), are positively related to achievement at standard significant levels. That is, the perception that one is learning, the perception that the evaluation method is right, and the sense that help is available when questions arise are the three key elements of teacher performance that matter most for student achievement. By contrast, the perception that their teacher motivates them (*AUTON_1*) and the provision of good materials (*STRUC_2*) are not directly linked to higher levels of student achievement. Moreover, how clearly the lecturer presented the syllabus (*STRUC_1*) is negatively related to achievement–which is rather surprising though it is good to note that this coefficient is of small magnitude and is statistically significant at a lower level (95%). We undertook several robustness checks that are briefly commented on in the supplementary material. See also Table D in S1 File.

Next, we considered whether students studying for different degrees may assign different importance to the three dimensions considered–that is, they feel different needs in terms of autonomy support, structure, involvement, or self-efficacy in order to reach their full potential in terms of achievement. To that end, we divided our sample into six subgroups– 1) Humanities, 2) Social Sciences, 3) Sciences, 4) Life Sciences, 5) Medical Sciences, and 6) Architecture and Engineering–and ran separate regressions for each of them, while accounting for all possible fixed effects (subject, year, and lecturer).

Table 3 presents a summary of the results by giving details on whether a statistically significant relationship was found and whether it was positive or negative. From the results, we learn that the positive correlation between *EFFICACY_2* and achievement is driven by all knowledge areas, as indicated by the positive coefficient in all the regressions with a confidence level of 99%. Results relative to *INVOL_1* are driven by students in Humanities, Social Sciences, and Architecture/Engineering. Thus, while all students appreciate being evaluated in a way that they feel allows them to show the knowledge acquired, the availability and relatedness of teachers matters most for these three groups. Students in Sciences, Life Sciences, and Medical Sciences do not feel the same need for teachers’ availability to achieve higher marks. Furthermore, results on *EFFICACY_1* are driven by most students, except for those in Humanities and Sciences (though one has to interpret such results cautiously, as they are drawn from a relatively small number of observations). The negative correlation found between *STRUC_1* and achievement is driven by students in Humanities and Architecture/Engineering (though, once again, at the 95% confidence level). Finally, it is worth pointing out that students in Humanities show a strong appreciation for autonomy support, as they are the only group for whom *AUTON_1* is positive and statistically significant at the 99% confidence level. In similar fashion, only among students in Architecture/Engineering does one find a positive relationship between achievement and *STRUC_2*, indicating that structure is important to them in the form of support materials that the teacher may provide. The detailed results of the regression outputs can be found in Table E in S1 File.

**Table 3:** Results from linear regression models with fixed effects (by subject, year, and lecturer) on achievement by knowledge areas.

	Humanities	Social Sciences	Sciences	Life Sciences	Medical Sciences	Architecture / Engineering
STRUC_1	[–] **					[–] **
STRUC_2						[+] **
AUTON_1	[+] ***					
INVOL_1	[+] **	[+] ***			[+] *	[+] **
EFFICACY_1		[+] ***		[+] ***	[+] ***	[+] ***
EFFICACY_2	[+] ***	[+] ***	[+] ***	[+] ***	[+] ***	[+] ***
Observations	3,852	47,496	1,530	6,929	12,760	13,439
R-squared	0.230	0.340	0.375	0.331	0.382	0.278

Note: Authors’ elaboration using teacher-evaluation questionnaires and students’ records at the University of Girona, 2014–2016. Level of significance:

*** p<0.01

** p<0.05

* p<0.1.

## Discussion

What matters for college students’ achievement is not so much *structure* or *autonomy support*, but rather the teacher’s *involvement* in the student learning process and students’ feelings of competence (*self-efficacy*), as measured by the impression students have that they are helped when questions arise, are learning, and are able to prove that they are learning. Our findings are relevant and novel in the context of higher education, as they provide new knowledge that contributes to a more nuanced understanding of the psychological needs of university students to improve their motivation and performance, and of the degree to which self-efficacy triggers the learning process. Previously, we were aware that the level of achievement correlates with students’ academic engagement [30], instructors’ teaching style [31, 32], and teachers’ professional competence [33, 34] and identity [35, 36]; but our results explain more precisely that achievement is promoted when students’ ability to recognize that they are learning is enhanced concomitantly with the feeling that lecturers are there to help [37].

Indeed, our study shows that high levels of self-efficacy predict better academic performance in all knowledge areas [38, 39]. Self-efficacy plays a predicting and mediating role in relation to students’ achievement, motivation, and learning [18]. In this sense, we regard as highly important the analysis of students’ *agentic engagement* [29]–that is, the students’ constructive contribution to the flow of instruction they receive from the teachers, because this is the concept that best captures student-initiated, proactive, intentional, collaborative, and constructive action [40]. But, while our results show that students’ self-efficacy correlates with academic adjustment, the small number of questions in the teacher-evaluation questionnaire available limits our analysis of the students’ responsiveness to personal-emotional adjustment, involving the way students come to terms with an environment characterized by attitudes and efforts. Given this substantial role, it would be relevant to gain a deeper insight into the year-on-year construction of students’ self-efficacy, and in particular see how higher education teachers could foster this continuous process.

In line with other studies [41], our results show that the three dimensions of NST are distinguishable by knowledge area; but our results do not support the hypothesis that there is an interplay among the NST dimensions to consistently predict students’ achievement (*1*). Multiple factors can explain why university students from different knowledge areas show different needs–factors such as individual abilities, intelligence, personal aptitude, interests, background, or the values that make up their belief system and personality [42]. Among Humanities students, for example, achievement is correlated with autonomy support and involvement; among those in Architecture and Engineering, the correlation is with structure and involvement; among those in Social and Medical Sciences, it is with involvement; and among those studying Life Sciences and Sciences there is no correlation with any of the NST dimensions. Thus, since students from different areas of knowledge present different psychological needs in relation to achievement, an in-depth study of these needs would result in strategies that can be used to plan and manage university teaching in the different areas of knowledge, if the objective is to provide more-effective teaching [43, 44]. And not only that: psychological needs should also be known and aligned with students’ individual profiles, in order to gain knowledge of the interrelationships between university students’ belief systems and their interpersonal influences [45, 46]. Such analyses were beyond the scope of this paper.

Getting to know college students better and gaining a better understanding of their needs will create more-supportive teachers, who can foster motivation and achievement among students in all areas of knowledge. Intervention-based research shows that teachers can learn how to become more supportive; but this same research also shows that teachers need considerable help and expert guidance to do so [47].

Our analysis is not without its limitations. First of all, as mentioned above, we were constrained by the nature and number of questions in the teacher-evaluation questionnaire. On top of that, we had no access to the accompanying text responses, which would have allowed a mixed-method analysis and a better understanding of the students’ quantitative answers. Second, we were also constrained by the number of controls in the specifications as, for example, neither age nor gender was provided to us. Third, since the questionnaire was completed voluntarily by the students, we do not know if our sample provides a good representation of all students at the university. Similarly, our results refer to higher education students, and so care should be taken about generalizing our findings for primary or secondary education. Finally, with the data to hand, we have been unable to determine the origin of the differences in the results by knowledge area. In this respect, it would have been useful to know beforehand the psychological needs of the students pursuing different degree courses. All these limitations open avenues for future research.

## Supporting information

S1 File Materials and methods, supplementary figure and tables. (DOCX)
