# Supplementary material for: Teachers’ involvement and students’ self-efficacy: Keys to achievement in higher education
Source: PLoS One. 2019 May 24;14(5):e0216865. doi: 10.1371/journal.pone.0216865 (PMC6534320; doi:10.1371/journal.pone.0216865)
Supplement: S1 File — (DOCX) [file pone.0216865.s001.docx]

Materials and Methods

**Materials**

Data used for our empirical analysis consist of the whole universe of teaching evaluations by students at the University of Girona from 2014 to 2016, as well as the marks they obtained per subject and academic year, which we draw from student records. Answers to all questions of the teacher evaluations are on a scale from 1 to 5, whereby 1 indicates “strong disagreement” and 5 “strong agreement”. Figure A. in this supplementary material details the frequency distribution for the answers to each of the questions; as is shown, these tend to be skewed to higher scores. Table A shows the mean of the answers by academic year; it can be seen that the means remain rather constant over time. As for more detail on achievement, Table B shows the average score obtained by students, conditional on their answers to each question in the teaching evaluation questionnaire. There is a positive direct relationship between agreeing strongly with the sentences of the questionnaire and obtaining a higher mark, although the differences are not that large between those who strongly disagree and those who strongly agree. It is important to highlight at this point that students answer the teaching evaluation questionnaire *before* they know their final mark in the subject.

Finally, Table C details the mean of the answers to the teaching evaluations and achievement by the six areas of knowledge considered in the paper. All the averages fit in the rather small range between 3.82 and 4.48, with a tendency for Architecture/Engineering students to disagree more strongly with the sentences of the questionnaire, and a tendency for students of Humanities to agree more strongly. Again, as for achievement, the students in Humanities are those who on average obtain the highest marks (followed by students in Medical Sciences and Social Sciences); those in Architecture or Engineering get the lowest. At this point, it is important to recall that the empirical strategy used in the paper avoids any bias that could derive from differences in the nature, difficulty, or type of evaluation that students reading for different degrees may receive.

**Methods**

*Formal specification of the econometric strategy*

Formally, our specifications take the following form:

$$A_{slit}=\alpha+\beta_{1}\cdot Q_{1slit}+\beta_{2}\cdot Q_{2slit}+\beta_{3}\cdot Q_{3slit}+\beta_{4}\cdot Q_{4slit}+\beta_{5}\cdot Q_{5slit}+\beta_{6}\cdot Q_{6slit}+S_{s}+L_{l}+T_{t}+\varepsilon$$

where $A_{slit}$is the final mark (achievement) obtained in subject *s*, with lecturer *l*, by student *i* during the academic year *t*, $\alpha$ is the constant term, and $\beta_{1}$ through $\beta_{6}$ are the main parameters of interest, as they are going to establish the extent to which students’ perceptions of their teacher support (expressed by their answers to the questionnaire) are related to their achievement. $S_{s}$ are the fixed effects by subject, $L_{l}$ are the fixed effects by lecturer, and $T_{t}$ are the fixed effects by academic year. Finally, $\varepsilon$ is the usual error term.

All the results have been computed using the software package Stata© 15.1 licensed to S.A.

*Robustness checks*

We undertook several robustness checks to confirm our findings. First, we ran the same regressions as those presented in Table 2 in the main text, by clustering standard errors. That is, we account for the possibility that there are unobserved factors that cause units to be correlated within clusters, for example, the learning environment within a subject [*48,* *49*]. In a first set of regressions, we cluster by course subject, and in a second set, by course subject and academic year. In all these auxiliary regressions, standard errors tend to increase slightly, but our findings remain unchanged. See columns A and B in Table D in this supplementary material.

Second, we considered exploiting (i) the discrete nature of our dependent variable, and (ii) the discrete nature of our explanatory variables. To that end, we ran a series of ordered probit models, as detailed in columns C and D in Table D. Again, results are qualitatively the same as those presented in Table 2 – the positive relationship between achievement and *INVOL_1* and *INVOL_2* are particularly reinforced when both the dependent and the explanatory variables are discrete.

**Figure A**. Frequency distribution for the answers to the teacher evaluation questionnaires.

Note: Answers to the questions are on a scale from 1 to 5, whereby 1 refers to “strong disagreement” and 5 to “strong agreement”. *STRUC_1*: This teacher has introduced the course syllabus and the evaluation criteria clearly. *STRUC_2*: The course support material that the teacher provides me with helps. *AUTON_1*: This teacher motivates me to make an effort and to learn by myself. *INVOL_1*: This teacher has helped me with my doubts when I consulted him/her. *EFFICACY_1*: With this teacher, I learn. *EFFICACY_2*: The evaluation procedure allows me to show my knowledge. Data are from teaching evaluations by students at the University of Girona (Spain) from 2014 to 2016. *N*=86,038 observations.

**Table A.** Mean of the answers to the teaching evaluations and achievement by year.

|  | **2014** | **2015** | **2016** |
| --- | --- | --- | --- |
| STRUC_1 | 4.18 | 4.23 | 4.20 |
| STRUC_2 | 3.91 | 3.96 | 3.93 |
| AUTON_1 | 3.77 | 3.83 | 3.80 |
| INVOL_1 | 4.26 | 4.34 | 4.30 |
| EFFICACY_1 | 3.99 | 4.04 | 4.01 |
| EFFICACY_2 | 3.81 | 3.87 | 3.82 |
| Achievement | 6.82 | 6.87 | 6.86 |

Note: Answers to the questions are on a scale from 1 to 5, whereby 1 refers to “strong disagreement” and 5 to “strong agreement”. *STRUC_1*: This teacher has introduced the course syllabus and the evaluation criteria clearly. *STRUC_2*: The course support material that the teacher provides me with helps. *AUTON_1*: This teacher motivates me to make an effort and to learn by myself. *INVOL_1*: This teacher has helped me with my doubts when I consulted him/her. *EFFICACY_1*: With this teacher, I learn. *EFFICACY_2*: The evaluation procedure allows me to show my knowledge. Data are from teaching evaluations by students at the University of Girona (Spain) from 2014 to 2016. *N*=86,038 observations.

**Table B.** Average score (achievement) obtained by students, conditional on their answers to each question of the teaching evaluation questionnaire.

| Answer | **STRUC_1** | **STRUC_2** | **AUTON_1** | **INVOL_1** | **EFFICACY_1** | **EFFICACY_2** |
| --- | --- | --- | --- | --- | --- | --- |
| 1 | 6.30 | 6.27 | 6.25 | 6.24 | 6.16 | 6.18 |
| 2 | 6.69 | 6.58 | 6.64 | 6.55 | 6.61 | 6.56 |
| 3 | 6.77 | 6.78 | 6.82 | 6.64 | 6.73 | 6.71 |
| 4 | 6.90 | 6.96 | 6.99 | 6.85 | 6.93 | 7.00 |
| 5 | 7.12 | 7.20 | 7.20 | 7.17 | 7.18 | 7.26 |

Note: Answers to the questions are on a scale from 1 to 5, whereby 1 refers to “strong disagreement” and 5 to “strong agreement”. *STRUC_1*: This teacher has introduced the course syllabus and the evaluation criteria clearly. *STRUC_2*: The course support material that the teacher provides me with helps. *AUTON_1*: This teacher motivates me to make an effort and to learn by myself. *INVOL_1*: This teacher has helped me with my doubts when I consulted him/her. *EFFICACY_1*: With this teacher, I learn. *EFFICACY_2*: The evaluation procedure allows me to show my knowledge. Data are from teaching evaluations by students at the University of Girona (Spain) from 2014 to 2016. *N*=86,038 observations.

**Table C.** Mean of the answers to the teaching evaluations and achievement by area of knowledge.

|  | Humanities | Social  Sciences | Sciences | Life Sciences | Medical  Sciences | Architecture/  Engineering |
| --- | --- | --- | --- | --- | --- | --- |
| STRUC_1 | 4.44 | 4.30 | 4.44 | 4.30 | 4.42 | 4.20 |
| STRUC_2 | 4.41 | 4.13 | 4.26 | 4.16 | 4.36 | 4.01 |
| AUTON_1 | 4.22 | 3.95 | 4.08 | 3.93 | 4.18 | 3.85 |
| INVOL_1 | 4.48 | 4.32 | 4.37 | 4.28 | 4.45 | 4.16 |
| EFFICACY_1 | 4.31 | 4.06 | 4.13 | 4.03 | 4.21 | 3.89 |
| EFFICACY_2 | 4.14 | 3.96 | 4.06 | 3.83 | 4.14 | 3.82 |
| Achievement | 7.28 | 7.10 | 6.62 | 6.98 | 7.20 | 6.36 |

Note: Answers to the questions are on a scale from 1 to 5, whereby 1 refers to “strong disagreement” and 5 to “strong agreement”. *STRUC_1*: This teacher has introduced the course syllabus and the evaluation criteria clearly. *STRUC_2*: The course support material that the teacher provides me with helps. *AUTON_1*: This teacher motivates me to make an effort and to learn by myself. *INVOL_1*: This teacher has helped me with my doubts when I consulted him/her. *EFFICACY_1*: With this teacher, I learn. *EFFICACY_2*: The evaluation procedure allows me to show my knowledge. Data are from teaching evaluations by students at the University of Girona (Spain) from 2014 to 2016. *N*=86,038 observations.

**Table D**. Results (coefficients) from ordered regression probit models with fixed effects (by subject, year, and lecturer) on achievement.

|  | (A)  Linear regression model (as in Table 2) with standard errors clustered by subject | (B)  Linear regression model (as in Table 2) with standard errors clustered by subject and year | (C)  Ordered probit model  (explanatory variables as continuous) | (D)  Ordered probit model  (explanatory variables as categorical) |
| --- | --- | --- | --- | --- |
| STRUC­­­_1 | -0.014** (0.007) | -0.014** (0.007) | -0.016*** (0.005) |  |
| [2] |  |  |  | 0.026 (0.028) |
| [3] |  |  |  | -0.006 (0.027) |
| [4] |  |  |  | -0.032 (0.027) |
| [5] |  |  |  | -0.042 (0.027) |
| STRUC_2 | 0.003 (0.007) | 0.003 (0.007) | 0.000 (0.005) |  |
| [2] |  |  |  | -0.003 (0.024) |
| [3] |  |  |  | 0.023 (0.024) |
| [4] |  |  |  | 0.022 (0.024) |
| [5] |  |  |  | 0.006 (0.025) |
| AUTON_1 | -0.002 (0.008) | -0.002 (0.008) | 0.003 (0.006) |  |
| [2] |  |  |  | 0.033 (0.024) |
| [3] |  |  |  | 0.045* (0.025) |
| [4] |  |  |  | 0.050*(0.026) |
| [5] |  |  |  | 0.039 (0.027) |
| INVOL_1 | 0.043*** (0.008) | 0.043*** (0.008) | 0.033*** (0.005) |  |
| [2] |  |  |  | 0.040 (0.027) |
| [3] |  |  |  | 0.015 (0.026) |
| [4] |  |  |  | 0.032 (0.026) |
| [5] |  |  |  | 0.110*** (0.026) |
| EFFICACY_1 | 0.084*** (0.010) | 0.084*** (0.009) | 0.064*** (0.006) |  |
| [2] |  |  |  | 0.116*** (0.026) |
| [3] |  |  |  | 0.101*** (0.028) |
| [4] |  |  |  | 0.177*** (0.029) |
| [5] |  |  |  | 0.256*** (0.031) |
| EFFICACY_2 | 0.119*** (0.008) | 0.119*** (0.007) | 0.097*** (0.005) |  |
| [2] |  |  |  | 0.092*** (0.022) |
| [3] |  |  |  | 0.161*** (0.021) |
| [4] |  |  |  | 0.295*** (0.021) |
| [5] |  |  |  | 0.373*** (0.022) |
| FE subject | Yes | Yes | Yes | Yes |
| FE year | Yes | Yes | Yes | Yes |
| FE lecturer | Yes | Yes | Yes | Yes |
| R-squared | 0.34 | 0.34 |  |  |
| (Pseudo)R-squared |  |  | 0.06 | 0.06 |
| Log-likelihood |  |  | -306902.3 | -306866.77 |

Note: *STRUC_1*: This teacher has introduced the course syllabus and the evaluation criteria clearly. *STRUC_2*: The course support material that the teacher provides me with helps. *AUTON_1*: This teacher motivates me to make an effort and to learn by myself. *INVOL_1*: This teacher has helped me with my doubts when I consulted him/her. *EFFICACY_1*: With this teacher, I learn. *EFFICACY_2*: The evaluation procedure allows me to show my knowledge. Column (A) details the results from a linear regression model with standard errors clustered by subject. Column (B) details the results from a linear regression model with standard errors clustered by subject and year. Column (C) details the results from an ordered probit model with the main explanatory variables as continuous. Column (D) details the results from an ordered probit model with the main explanatory variables as categorical. Authors’ elaboration using teacher-evaluation questionnaires at the University of Girona, from 2014 to 2016. Standard errors in parentheses. *N*=86,038 observations. Level of significance: *** p<0.01; ** p<0.05; * p<0.1.

**Table E**. Results (coefficients) from linear regression models with fixed effects (by subject, year, and lecturer) on achievement by areas of knowledge.

|  | Humanities | Social Sciences | Sciences | Life Sciences | Medical Sciences | Architecture / Engineering |
| --- | --- | --- | --- | --- | --- | --- |
| STRUC_1 | -0.0824** | -0.0055 | 0.0582 | -0.0261 | -0.0095 | -0.0400** |
|  | (0.0378) | (0.0084) | (0.0641) | (0.0241) | (0.0163) | (0.0191) |
| STRUC_2 | -0.0278 | -0.0062 | -0.0034 | -0.0159 | 0.0070 | 0.0393** |
|  | (0.0386) | (0.0084) | (0.0605) | (0.0219) | (0.0144) | (0.0190) |
| AUTON_1 | 0.1153*** | 0.0001 | -0.0321 | 0.0144 | -0.0059 | -0.0335 |
|  | (0.0425) | (0.0091) | (0.0698) | (0.0248) | (0.0167) | (0.0211) |
| INVOL_1 | 0.0934** | 0.0496*** | -0.0535 | 0.0081 | 0.0277* | 0.0475** |
|  | (0.0398) | (0.0087) | (0.0651) | (0.0236) | (0.0164) | (0.0205) |
| EFFICACY_1 | 0.0360 | 0.0770*** | 0.1261 | 0.1014*** | 0.0634*** | 0.1205*** |
|  | (0.0492) | (0.0104) | (0.0819) | (0.0283) | (0.0197) | (0.0242) |
| EFFICACY_2 | 0.1589*** | 0.1133*** | 0.1677*** | 0.1638*** | 0.0724*** | 0.1390*** |
|  | (0.0345) | (0.0078) | (0.0531) | (0.0190) | (0.0135) | (0.0178) |
| Observations | 3,852 | 47,496 | 1,530 | 6,929 | 12,760 | 13,439 |
| R-squared | 0.2296 | 0.3400 | 0.3748 | 0.3305 | 0.3819 | 0.2777 |

Note. *STRUC_1*: This teacher has introduced the course syllabus and the evaluation criteria clearly. *STRUC_2*: The course support material that the teacher provides me with helps. *AUTON_1*: This teacher motivates me to make an effort and to learn by myself. *INVOL_1*: This teacher has helped me with my doubts when I consulted him/her. *EFFICACY_1*: With this teacher, I learn. *EFFICACY_2*: The evaluation procedure allows me to show my knowledge. Authors’ elaboration using teacher-evaluation questionnaires at the University of Girona, from 2014 to 2016. Standard errors in parentheses. *N*=86,038 observations. Level of significance: *** p<0.01; ** p<0.05; * p<0.1.

References

48. A. C. Cameron, D. L. Miller, A practitioner’s guide to cluster-robust inference. *J. Hum. Resour.* **50**, 317–372 (2015). doi: 10.3368/jhr.50.2.317.

49. A. Abadie, S. Athey, G. W. Imbens, J. Wooldridge, “When should you adjust standard errors for clustering?” (WP 24003, NBER, 2017).
